# *N*,*N*′-*p*-Phenyl­enediisonicotinamide monohydrate

**DOI:** 10.1107/S1600536809024684

**Published:** 2009-07-04

**Authors:** Li Song, Wenxiang Chai, Junwei Lan

**Affiliations:** a Department of Chemistry, Key Laboratory of Advanced Textile Materials and Manufacturing Technology of the Ministry of Education, Zhejiang Sci-Tech University, Hangzhou 310018, People’s Republic of China; b College of Materials Science and Engineering, China Jiliang University, Hangzhou 310018, People’s Republic of China

## Abstract

The organic mol­ecule of the title compound, C_18_H_14_N_4_O_2_·H_2_O, lies on a center of inversion located at the centre of the central phenyl­ene ring. There are two half-molecules in the asymmetric unit. In the crystal, the mol­ecules are linked through by N—H⋯O and O—H⋯N hydrogen bonds involving the water mol­ecule, forming a layer structure. The layers inter­act by π–π inter­actions between the aromatic rings.

## Related literature

For background to *N*,*N*′-*p*-phenylenediisonicotinamide complexes, see: Burchell *et al.* (2003 ▶, 2004 ▶); Niu *et al.* (2004 ▶); Pansanel *et al.* (2006 ▶).
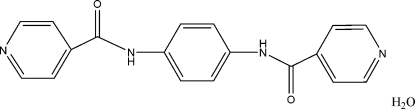


## Experimental

### 

#### Crystal data

C_18_H_14_N_4_O_2_·H_2_O
*M* *_r_* = 336.35
Triclinic, 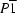

*a* = 6.9936 (14) Å
*b* = 10.852 (2) Å
*c* = 11.285 (2) Å
α = 95.98 (3)°
β = 106.36 (3)°
γ = 94.68 (3)°
*V* = 811.8 (3) Å^3^
*Z* = 2
Mo *K*α radiation
μ = 0.10 mm^−1^
*T* = 296 K
0.32 × 0.21 × 0.13 mm

#### Data collection

Rigaku R-AXIS RAPID diffractometer
Absorption correction: multi-scan (*CrystalStructure*; Rigaku/MSC, 2004 ▶) *T*_min_ = 0.976, *T*_max_ = 0.987
7994 measured reflections
3671 independent reflections
1782 reflections with *I* > 2σ(*I*)
*R*_int_ = 0.039

#### Refinement

*R*[*F* ^2^ > 2σ(*F* ^2^)] = 0.050
*wR*(*F* ^2^) = 0.163
*S* = 1.10
3671 reflections
235 parameters
H atoms treated by a mixture of independent and constrained refinement
Δρ_max_ = 0.24 e Å^−3^
Δρ_min_ = −0.20 e Å^−3^

### 

Data collection: *PROCESS-AUTO* (Rigaku, 1998 ▶); cell refinement: *PROCESS-AUTO*; data reduction: *CrystalStructure* (Rigaku/MSC, 2004 ▶); program(s) used to solve structure: *SHELXS97* (Sheldrick, 2008 ▶); program(s) used to refine structure: *SHELXL97* (Sheldrick, 2008 ▶); molecular graphics: *SHELXTL* (Sheldrick, 2008 ▶); software used to prepare material for publication: *SHELXTL*.

## Supplementary Material

Crystal structure: contains datablocks I, global. DOI: 10.1107/S1600536809024684/ng2603sup1.cif

Structure factors: contains datablocks I. DOI: 10.1107/S1600536809024684/ng2603Isup2.hkl

Additional supplementary materials:  crystallographic information; 3D view; checkCIF report

## Figures and Tables

**Table 1:** Hydrogen-bond geometry (Å, °)

*D*—H⋯*A*	*D*—H	H⋯*A*	*D*⋯*A*	*D*—H⋯*A*
N2—H2*A*⋯O3^i^	0.88	2.00	2.847 (3)	160
N4—H4*A*⋯O1	0.88	2.12	2.968 (3)	161
O3—H15⋯N1	0.94 (4)	1.92 (4)	2.845 (3)	168 (3)
O3—H16⋯N3^ii^	0.87 (4)	2.01 (4)	2.849 (3)	162 (4)

Symmetry codes: (i) 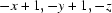; (ii) 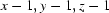.

**Fig. 1.**
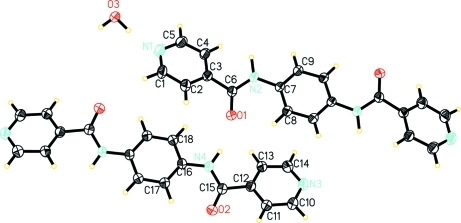
Structure and labeling of compound 1, with displacement ellipsoids drawn at the 30% probability level and H atoms shown as small spheres of arbitrary radii.

**Fig. 2.**
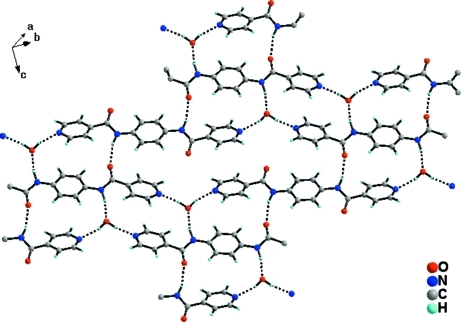
The layer formed through the intermolecular hydrogen bonds.

**Fig. 3.**
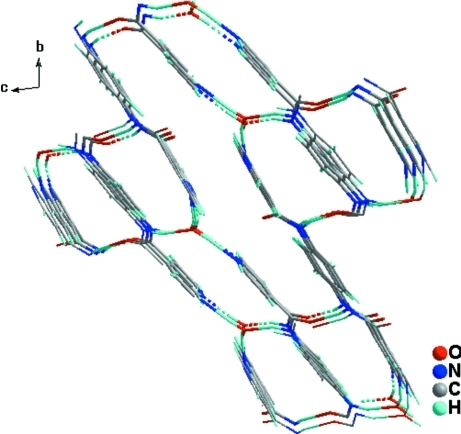
The packing diagram viewed along the a-direction.

## supplementary crystallographic information

### Comment

The incorporation of amide groups into organic ligands are of interests because of the existence of great and typical intermolecular hydrogen bondings. Furthermore, the bridging bis(pyridyl) ligands are good donors for building metal-organic frameworks (MOFs). As one example of bis(pyridyl) ligands with amide groups, *N*,*N*'-(biphenyl-4,4'-diyl)diisonicotinamide has been very less studied. Only a few examples built upon *N*,*N*'-(biphenyl-4,4'-diyl)diisonicotinamide and Cu(II), Hg(I) salts have been reported. However, the crystal structure of the ligand, *N*,*N*'-(biphenyl-4,4'-diyl)diisonicotinamide, has not been described in detail. Herein, we report the crystal structure and characterization of the title compound *N*,*N*'-(biphenyl-4,4'-diyl)diisonicotinamide.

The compound crystallizes in triclinic form in the space group P-1. As shown in Figure 1, the title compound, C18H14N4O2.H2O, contains an *N*,*N*'-(biphenyl-4,4'-diyl)diisonicotinamide molecule and a water solvent molecule. The bond lengths of the two independent parts of the C18H14N4O2 molecule display slight differences. The two C=O bonds of the amide groups are 1.223 (3) Å and 1.232 (3) Å long respectively. Individual molecules are connected through intermolecular N—H···O=C hydrogen bonds between amide groups The H(4 A)···O(1) distance is 2.12 Å and the N(4)···O(1) distance is 2.968 (3) Å. The N(4)—H(4 A)···O(1) angle is 161.3 °.

As shown in Figure 2, The molecules and the solvent water molecules are connected through the O—H···N hydrogen bonds between the water molecules and the pyridine groups or the amide groups (N(2)···O(3) = 2.847 (3) Å, O(3)···N(1) = 2.845 (3) Å, O(3)···N(3) = 2.849 (3) Å) to form a layer. The hydrogen bond geometry is listed in table 1. The layers pack *via*π-π interactions among the phenyl rings (Figure 3).

### Experimental

The synthesis of compound 1 was modified with reference to the literature methods (*R*. J. Puddephatt and H. W. Hou). Isonicotinic acid (4.924 g, 40.0 mmol) was refluxed in thionyl chloride (20 ml) for 4 h. Excess thionyl chloride was removed under vacuum leaving a colorless solid. The solid was suspended in tetrahydrofuran (80 ml) and then a solution of 1,4-phenylenediamine (1.622 g, 0.015 mol) in tetrahydrofuran (20 ml) was added. After 15 minutes of stirring, triethylamine (15.0 ml) was added. The solution was refluxed at 70 ?C for 6 h and cooled to room temperature. Removal of the excess solvents results in a great deal of light-yellow solid. A solution of K2CO3 (6.910 g, 50 mmol) in water (40 ml) was added into the solid. After 10 minutes of stirring, the white products were filtered, washed with water and ethanol. Yield: *ca* 82%. Well shaped colorless crystals were obtained by slow evaporation of DMF/THF solution. 1H NMR (400 MHz, DMSO): ^1^H 7.781 (s, 4H), 7.879 (d, 4H), 8.786 (d, 4H), 10.527 (s, 2H). Anal. Calcd for C18H16N4O3: C 64.28, H 4.79, N 16.66. Found (%): C 64.12, H 4.91, N 16.78. IR (KBr pellet, cm^-1^): 3329(*s*), 1647(*s*), 1611(*m*), 1593(sh), 1547(*s*), 1522(*s*), 1506(sh), 1485(w), 1413(*m*), 1321(*m*), 1276(w), 1219(w), 1066(w), 827(*m*), 756(w), 667(*m*).

### Refinement

The structure was solved using direct methods and refined by full-matrix least-squares techniques. All non-hydrogen atoms were assigned anisotropic displacement parameters in the refinement. All hydrogen atoms were added at calculated positions and refined using a riding model, except that two hydrogen atoms of the solvent water were picked out by Difference Fourier Syntheses. The structure was refined on F2 using *SHELXTL97* software package(Sheldrick *et al.*, 2008) without any unusual events.

### Crystal data

**Table:**

C_18_H_14_N_4_O_2_·H_2_O	*Z* = 2
*M**_r_* = 336.35	*F*(000) = 352
Triclinic, *P*1	*D*_x_ = 1.376 Mg m^−^^3^
*a* = 6.9936 (14) Å	Mo *K*α radiation, λ = 0.71073 Å
*b* = 10.852 (2) Å	θ = 3.1–27.5°
*c* = 11.285 (2) Å	µ = 0.10 mm^−^^1^
α = 95.98 (3)°	*T* = 296 K
β = 106.36 (3)°	Block, colorless
γ = 94.68 (3)°	0.32 × 0.21 × 0.13 mm
*V* = 811.8 (3) Å^3^	

### Data collection

**Table:**

Rigaku R-AXIS RAPID diffractometer	3671 independent reflections
Radiation source: fine-focus sealed tube	1782 reflections with *I* > 2σ(*I*)
Graphite Monochromator	*R*_int_ = 0.039
ω scans	θ_max_ = 27.5°, θ_min_ = 3.1°
Absorption correction: multi-scan (*CrystalStructure*; Rigaku/MSC, 2004)	*h* = −9→8
*T*_min_ = 0.976, *T*_max_ = 0.987	*k* = −14→13
7994 measured reflections	*l* = −14→14

### Refinement

**Table:**

Refinement on *F*^2^	Secondary atom site location: difference Fourier map
Least-squares matrix: full	Hydrogen site location: inferred from neighbouring sites
*R*[*F*^2^ > 2σ(*F*^2^)] = 0.050	H atoms treated by a mixture of independent and constrained refinement
*wR*(*F*^2^) = 0.163	*w* = 1/[σ^2^(*F*_o_^2^) + (0.0641*P*)^2^ + 0.0836*P*] where *P* = (*F*_o_^2^ + 2*F*_c_^2^)/3
*S* = 1.10	(Δ/σ)_max_ < 0.001
3671 reflections	Δρ_max_ = 0.24 e Å^−^^3^
235 parameters	Δρ_min_ = −0.20 e Å^−^^3^
0 restraints	Extinction correction: *SHELXL97* (Sheldrick, 2008), Fc^*^=kFc[1+0.001xFc^2^λ^3^/sin(2θ)]^-1/4^
Primary atom site location: structure-invariant direct methods	Extinction coefficient: 0.014 (3)

### Special details

**Table:**

Geometry. All e.s.d.'s (except the e.s.d. in the dihedral angle between two l.s. planes) are estimated using the full covariance matrix. The cell e.s.d.'s are taken into account individually in the estimation of e.s.d.'s in distances, angles and torsion angles; correlations between e.s.d.'s in cell parameters are only used when they are defined by crystal symmetry. An approximate (isotropic) treatment of cell e.s.d.'s is used for estimating e.s.d.'s involving l.s. planes.
Refinement. Refinement of *F*^2^ against ALL reflections. The weighted *R*-factor *wR* and goodness of fit *S* are based on *F*^2^, conventional *R*-factors *R* are based on *F*, with *F* set to zero for negative *F*^2^. The threshold expression of *F*^2^ > σ(*F*^2^) is used only for calculating *R*-factors(gt) *etc*. and is not relevant to the choice of reflections for refinement. *R*-factors based on *F*^2^ are statistically about twice as large as those based on *F*, and *R*- factors based on ALL data will be even larger.

### Fractional atomic coordinates and isotropic or equivalent isotropic displacement parameters (Å^2^)

**Table:**

	*x*	*y*	*z*	*U*_iso_*/*U*_eq_	
O1	0.5143 (3)	0.75658 (16)	0.44339 (18)	0.0695 (6)	
N1	0.3107 (4)	0.4128 (2)	0.0888 (2)	0.0784 (8)	
C1	0.2062 (5)	0.4882 (3)	0.1391 (3)	0.0791 (9)	
H1	0.0645	0.4691	0.1150	0.095*	
O2	0.1628 (3)	0.77553 (17)	0.78063 (19)	0.0773 (6)	
N2	0.7556 (3)	0.79239 (16)	0.34696 (18)	0.0511 (5)	
H2A	0.7946	0.7654	0.2822	0.061*	
C2	0.2908 (4)	0.5924 (2)	0.2241 (3)	0.0667 (7)	
H2	0.2090	0.6436	0.2569	0.080*	
O3	0.1253 (3)	0.2348 (2)	−0.12516 (19)	0.0774 (7)	
N3	0.7983 (4)	1.0595 (2)	0.8737 (2)	0.0693 (7)	
C3	0.4971 (4)	0.6211 (2)	0.2608 (2)	0.0507 (6)	
N4	0.2782 (3)	0.70930 (17)	0.61858 (19)	0.0545 (5)	
H4A	0.3716	0.7266	0.5825	0.065*	
C4	0.6076 (4)	0.5436 (2)	0.2091 (2)	0.0557 (7)	
H4	0.7495	0.5607	0.2313	0.067*	
C5	0.5096 (5)	0.4414 (2)	0.1251 (3)	0.0680 (8)	
H5	0.5877	0.3882	0.0911	0.082*	
C6	0.5894 (4)	0.7301 (2)	0.3589 (2)	0.0522 (6)	
C7	0.8750 (3)	0.89703 (19)	0.4275 (2)	0.0458 (6)	
C8	0.8041 (4)	0.9772 (2)	0.5031 (2)	0.0558 (7)	
H8	0.6698	0.9625	0.5058	0.067*	
C9	1.0706 (4)	0.9205 (2)	0.4249 (2)	0.0556 (6)	
H9	1.1201	0.8653	0.3725	0.067*	
C10	0.6170 (4)	1.0792 (2)	0.8812 (3)	0.0671 (8)	
H10	0.6015	1.1572	0.9219	0.081*	
C11	0.4505 (4)	0.9927 (2)	0.8333 (2)	0.0582 (7)	
H11	0.3243	1.0105	0.8426	0.070*	
C12	0.4690 (4)	0.8799 (2)	0.7717 (2)	0.0517 (6)	
C13	0.6558 (4)	0.8578 (2)	0.7638 (3)	0.0612 (7)	
H13	0.6748	0.7810	0.7224	0.073*	
C14	0.8155 (4)	0.9488 (3)	0.8169 (3)	0.0712 (8)	
H14	0.9447	0.9316	0.8128	0.085*	
C15	0.2873 (4)	0.7842 (2)	0.7236 (3)	0.0558 (6)	
H15	0.173 (6)	0.289 (3)	−0.050 (4)	0.135 (14)*	
C16	0.1331 (3)	0.6052 (2)	0.5602 (2)	0.0483 (6)	
H16	0.026 (6)	0.191 (4)	−0.110 (4)	0.130 (14)*	
C17	−0.0548 (4)	0.5897 (2)	0.5761 (2)	0.0569 (7)	
H17	−0.0939	0.6510	0.6283	0.068*	
C18	0.1870 (4)	0.5146 (2)	0.4836 (2)	0.0563 (7)	
H18	0.3165	0.5244	0.4720	0.068*	

### Atomic displacement parameters (Å^2^)

**Table:**

	*U*^11^	*U*^22^	*U*^33^	*U*^12^	*U*^13^	*U*^23^
O1	0.0723 (13)	0.0736 (12)	0.0619 (12)	−0.0209 (9)	0.0365 (11)	−0.0191 (9)
N1	0.101 (2)	0.0679 (15)	0.0548 (16)	−0.0267 (14)	0.0189 (15)	−0.0060 (12)
C1	0.070 (2)	0.091 (2)	0.0567 (19)	−0.0302 (16)	0.0055 (16)	−0.0115 (16)
O2	0.0662 (13)	0.0869 (13)	0.0788 (15)	−0.0163 (10)	0.0395 (11)	−0.0206 (10)
N2	0.0530 (12)	0.0506 (11)	0.0433 (12)	−0.0092 (9)	0.0136 (9)	−0.0091 (8)
C2	0.0580 (17)	0.0734 (17)	0.0593 (18)	−0.0111 (13)	0.0120 (14)	−0.0043 (14)
O3	0.0852 (15)	0.0810 (13)	0.0592 (13)	−0.0318 (11)	0.0332 (12)	−0.0226 (10)
N3	0.0677 (16)	0.0679 (14)	0.0659 (16)	−0.0140 (11)	0.0201 (13)	−0.0044 (12)
C3	0.0562 (16)	0.0519 (13)	0.0396 (14)	−0.0060 (11)	0.0122 (12)	0.0012 (10)
N4	0.0539 (13)	0.0534 (11)	0.0528 (13)	−0.0095 (9)	0.0181 (10)	−0.0038 (9)
C4	0.0661 (17)	0.0486 (13)	0.0466 (15)	−0.0039 (11)	0.0126 (13)	−0.0002 (11)
C5	0.094 (2)	0.0542 (15)	0.0542 (17)	−0.0023 (14)	0.0240 (16)	0.0021 (12)
C6	0.0523 (15)	0.0524 (14)	0.0472 (15)	−0.0070 (11)	0.0130 (12)	0.0000 (11)
C7	0.0471 (14)	0.0471 (12)	0.0387 (13)	−0.0033 (10)	0.0110 (11)	−0.0035 (10)
C8	0.0473 (14)	0.0605 (14)	0.0546 (16)	−0.0063 (11)	0.0176 (12)	−0.0122 (12)
C9	0.0536 (15)	0.0536 (13)	0.0558 (16)	−0.0037 (11)	0.0198 (13)	−0.0126 (11)
C10	0.071 (2)	0.0572 (15)	0.0680 (19)	−0.0055 (13)	0.0215 (16)	−0.0059 (13)
C11	0.0575 (16)	0.0543 (14)	0.0620 (17)	0.0015 (11)	0.0206 (14)	−0.0006 (12)
C12	0.0530 (16)	0.0498 (13)	0.0489 (15)	−0.0001 (10)	0.0123 (12)	0.0032 (11)
C13	0.0532 (16)	0.0585 (15)	0.0693 (19)	−0.0023 (12)	0.0219 (14)	−0.0072 (13)
C14	0.0575 (18)	0.0773 (18)	0.075 (2)	−0.0038 (14)	0.0213 (15)	−0.0022 (15)
C15	0.0544 (16)	0.0547 (14)	0.0558 (17)	−0.0018 (11)	0.0171 (13)	−0.0006 (12)
C16	0.0444 (14)	0.0488 (13)	0.0488 (15)	−0.0018 (10)	0.0121 (11)	0.0030 (11)
C17	0.0500 (15)	0.0582 (14)	0.0600 (17)	−0.0005 (11)	0.0188 (13)	−0.0058 (12)
C18	0.0470 (15)	0.0604 (15)	0.0599 (17)	−0.0030 (11)	0.0194 (13)	−0.0031 (12)

### Geometric parameters (Å, °)

**Table:**

O1—C6	1.232 (3)	C5—H5	0.9500
N1—C1	1.327 (4)	C7—C8	1.372 (3)
N1—C5	1.335 (4)	C7—C9	1.380 (3)
C1—C2	1.375 (4)	C8—C9^i^	1.383 (3)
C1—H1	0.9500	C8—H8	0.9500
O2—C15	1.223 (3)	C9—C8^i^	1.383 (3)
N2—C6	1.343 (3)	C9—H9	0.9500
N2—C7	1.420 (3)	C10—C11	1.375 (3)
N2—H2A	0.8800	C10—H10	0.9500
C2—C3	1.383 (3)	C11—C12	1.377 (3)
C2—H2	0.9500	C11—H11	0.9500
O3—H15	0.94 (4)	C12—C13	1.373 (3)
O3—H16	0.87 (4)	C12—C15	1.506 (3)
N3—C10	1.327 (3)	C13—C14	1.378 (4)
N3—C14	1.333 (3)	C13—H13	0.9500
C3—C4	1.379 (3)	C14—H14	0.9500
C3—C6	1.498 (3)	C16—C17	1.375 (3)
N4—C15	1.348 (3)	C16—C18	1.387 (3)
N4—C16	1.422 (3)	C17—C18^ii^	1.382 (3)
N4—H4A	0.8800	C17—H17	0.9500
C4—C5	1.375 (3)	C18—C17^ii^	1.382 (3)
C4—H4	0.9500	C18—H18	0.9500
			
C1—N1—C5	116.7 (2)	C9^i^—C8—H8	120.2
N1—C1—C2	124.0 (3)	C7—C9—C8^i^	121.2 (2)
N1—C1—H1	118.0	C7—C9—H9	119.4
C2—C1—H1	118.0	C8^i^—C9—H9	119.4
C6—N2—C7	127.0 (2)	N3—C10—C11	123.5 (2)
C6—N2—H2A	116.5	N3—C10—H10	118.2
C7—N2—H2A	116.5	C11—C10—H10	118.2
C1—C2—C3	118.8 (3)	C10—C11—C12	119.2 (2)
C1—C2—H2	120.6	C10—C11—H11	120.4
C3—C2—H2	120.6	C12—C11—H11	120.4
H15—O3—H16	100 (3)	C13—C12—C11	118.0 (2)
C10—N3—C14	116.8 (2)	C13—C12—C15	123.2 (2)
C4—C3—C2	117.9 (2)	C11—C12—C15	118.7 (2)
C4—C3—C6	123.4 (2)	C12—C13—C14	119.0 (2)
C2—C3—C6	118.6 (2)	C12—C13—H13	120.5
C15—N4—C16	126.6 (2)	C14—C13—H13	120.5
C15—N4—H4A	116.7	N3—C14—C13	123.5 (3)
C16—N4—H4A	116.7	N3—C14—H14	118.3
C5—C4—C3	119.1 (3)	C13—C14—H14	118.3
C5—C4—H4	120.5	O2—C15—N4	124.2 (2)
C3—C4—H4	120.5	O2—C15—C12	120.4 (2)
N1—C5—C4	123.5 (3)	N4—C15—C12	115.4 (2)
N1—C5—H5	118.2	C17—C16—C18	118.9 (2)
C4—C5—H5	118.2	C17—C16—N4	123.8 (2)
O1—C6—N2	124.6 (2)	C18—C16—N4	117.3 (2)
O1—C6—C3	120.3 (2)	C16—C17—C18^ii^	120.2 (2)
N2—C6—C3	115.1 (2)	C16—C17—H17	119.9
C8—C7—C9	119.1 (2)	C18^ii^—C17—H17	119.9
C8—C7—N2	123.6 (2)	C17^ii^—C18—C16	120.8 (2)
C9—C7—N2	117.2 (2)	C17^ii^—C18—H18	119.6
C7—C8—C9^i^	119.6 (2)	C16—C18—H18	119.6
C7—C8—H8	120.2		

Symmetry codes: (i) −*x*+2, −*y*+2, −*z*+1; (ii) −*x*, −*y*+1, −*z*+1.

### Hydrogen-bond geometry (Å, °)

**Table:**

*D*—H···*A*	*D*—H	H···*A*	*D*···*A*	*D*—H···*A*
N2—H2A···O3^iii^	0.88	2.00	2.847 (3)	160
N4—H4A···O1	0.88	2.12	2.968 (3)	161
O3—H15···N1	0.94 (4)	1.92 (4)	2.845 (3)	168 (3)
O3—H16···N3^iv^	0.87 (4)	2.01 (4)	2.849 (3)	162 (4)

Symmetry codes: (iii) −*x*+1, −*y*+1, −*z*; (iv) *x*−1, *y*−1, *z*−1.

## References

[bb1] Burchell, T. J., Eisler, D. J., Jennings, M. C. & Puddephatt, R. J. (2003). *Chem. Commun.* pp. 2228–2229. 10.1039/b306004g 13678214

[bb2] Burchell, T. J., Eisler, D. J. & Puddephatt, R. J. (2004). *Inorg. Chem.* **43**, 5550–5557. 10.1021/ic049500+ 15332806

[bb3] Niu, Y. Y., Song, Y. L., Wu, J., Hou, H. W., Zhu, Y. & Wang, X. (2004). *Inorg. Chem. Commun.* **7**, 471–474.

[bb4] Pansanel, J., Jouaiti, A., Ferlay, S., Hosseini, M. W., Planeix, J. M. & Kyritsakas, N. (2006). *New J. Chem.* **30**, 71–76.

[bb5] Rigaku (1998). *PROCESS-AUTO* Rigaku Corporation, Tokyo, Japan.

[bb6] Rigaku/MSC (2004). *CrystalStructure* Rigaku/MSC, The Woodlands, Texas, USA.

[bb7] Sheldrick, G. M. (2008). *Acta Cryst.* A**64**, 112–122. 10.1107/S0108767307043930 18156677

